# Swift Assembly of Adaptive Thermocell Arrays for Device-Level Healable and Energy-Autonomous Motion Sensors

**DOI:** 10.1007/s40820-023-01170-x

**Published:** 2023-08-11

**Authors:** Xin Lu, Daibin Xie, Kaihua Zhu, Shouhao Wei, Ziwei Mo, Chunyu Du, Lirong Liang, Guangming Chen, Zhuoxin Liu

**Affiliations:** 1 College of Materials Science and Engineering, Shenzhen University, Shenzhen, 518055 People’s Republic of China; 2 State Key Laboratory of Polymer Materials Engineering, Sichuan University, Chengdu, 610065 People’s Republic of China

**Keywords:** Thermocells, Flexible devices, Wearable applications, Low-grade heat harvest, MXenes

## Abstract

The evolution of wearable technology has prompted the need for adaptive, self-healable, and energy-autonomous energy devices. This study innovatively addresses this challenge by introducing an MXene-boosted hydrogel electrolyte, which expedites the assembly process of flexible thermocell (TEC) arrays and thus circumvents the complicated fabrication of typical wearable electronics. Our findings underscore the hydrogel electrolyte's superior thermoelectrochemical performance under substantial deformations and repeated self-healing cycles. The resulting hydrogel-based TEC yields a maximum power output of 1032.1 nW under the Δ*T* of 20 K when being stretched to 500% for 1000 cycles, corresponding to 80% of its initial state; meanwhile, it sustains 1179.1 nW under the Δ*T* of 20 K even after 60 cut-healing cycles, approximately 92% of its initial state. The as-assembled TEC array exhibits device-level self-healing capability and high adaptability to human body. It is readily applied for touch-based encrypted communication where distinct voltage signals can be converted into alphabet letters; it is also employed as a self-powered sensor to in-situ monitor a variety of body motions for complex human actions. The swift assembly approach, combined with the versatile functionality of the TEC device, paves the way for future advancements in wearable electronics targeting at fitness monitoring and human–machine interfaces.

**Supplementary Information:**

The online version contains supplementary material available at 10.1007/s40820-023-01170-x.

## Highlights

- The MXene-boosted rapid gelling expedites the assembly of flexible thermocell arrays, overcoming the typical constraint of complicated device fabrication processes.
- The hydrogel electrolyte can sustain stable thermoelectrochemical performance under various challenging conditions, including large, repeated, and sustained deformations, and multiple cut-healing cycles.
- The as-assembled thermocell array exhibits device-level self-healing capability and high adaptability to human body, efficiently harvesting low-grade heat for wearable applications.

**Supplementary Information:**

The online version contains supplementary material available at 10.1007/s40820-023-01170-x.

## Graphical abstract

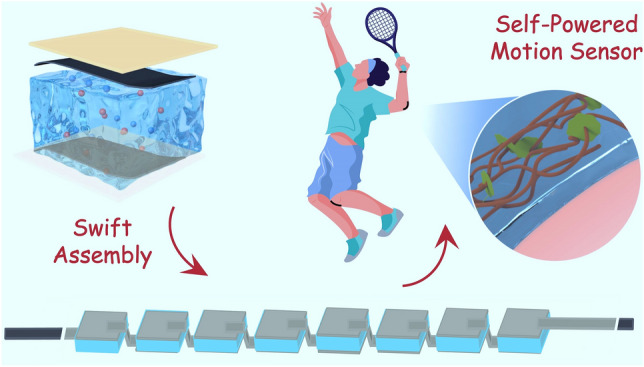

## Introduction

Flexible and wearable electronics have emerged as a rapidly growing field, offering unprecedented convenience and functionality in applications including fitness tracking, health monitoring, and human–machine interfaces [1–5]. Advancements in materials science, nanotechnology, and engineering have enabled the miniaturization of electronic components and the fabrication of thin, stretchable, and lightweight devices [6–8]. As the demand for wearable electronics continues to rise, so does the need for consistent, reliable, and flexible energy systems to power these devices. Batteries, solar cells, and triboelectric generators, while effective for many applications, require frequent charging, replacement, or can only function periodically [9–11]. In contrast, heat-to-electricity conversion techniques, which entail converting ambient thermal energy into usable electrical power, have emerged as a promising energy solution for energy-autonomous electronics [12–15]. These techniques offer the opportunity for continuous, self-sustained operation, reducing or even eliminating the need for external power supply.

Traditional thermoelectrics can realize the direct conversion between heat and electricity and have achieved much progress in recent years. However, the widely studied inorganic thermoelectric materials suffer from intrinsic rigidity/brittleness and bio-toxicity, making them unsuitable for flexible and wearable applications [16–18]. Although feasible solutions have been proposed to improve their usability by employing flexible substrates and highly integrated configurations, these approaches rely too much on precise micro-machining technology, resulting in high device manufacturing cost [19–21]. Organic thermoelectric materials, on the other hand, could be intrinsically soft and stretchable, while their thermopower are usually unsatisfactory [22, 23]. This limitation necessitates connecting and integrating thousands of thermoelectric legs for practical applications, thereby increasing both material and fabrication costs.

In contrast, thermocells (TECs) can generate a large thermoelectrochemical Seebeck coefficient (*S*_e_) via the thermogalvanic effect, which arises from the temperature-dependent redox reactions occurring at electrodes. Differing from the electronic Seebeck effect governed by electron/hole transport properties in traditional thermoelectrics, the redox ion-driven thermogalvanic effect leads to a considerable thermopower on the order of mV K^−1^ at ambient temperature, making TECs particularly suitable for low-grade heat harvest [24–26]; meanwhile, the recent development of quasi-solid-state hydrogel electrolytes for TECs, as a replacement for liquid electrolytes, can essentially address the leakage and encapsulation issues while providing solid-like, mechanically adaptable properties [27–31]. Thus, the inherent softness and conformability allow hydrogels to maintain close contact with human body and adapt to its movements; this attribute is essential for devices that require intimate integration with the wearer's skin to function effectively. Also, hydrogels generally exhibit lower thermal conductivities compared to traditional thermoelectric materials, which helps to maintain a consistent temperature gradient that is crucial for efficient energy conversion [32]. These distinctive merits render hydrogel-based TECs a promising energy solution for flexible and wearable electronics.

Several studies have successfully incorporated hydrogel electrolytes into advanced TEC devices. However, these investigations generally followed the device configuration of traditional thermoelectric generators, resulting in rigid device bodies that offset the merit of hydrogel conformability to accommodate human body motions [33–36]. While some flexible, deformable, and stretchable TECs have been explored, these devices necessitate either intricate hydrogel synthesis or complex device assembly procedures, limiting their scalability and affordability [37, 38]. Furthermore, these TECs lack device-level self-healing capabilities to recover from external mechanical damages. Hence, it is crucial to equip TECs with self-healing abilities, particularly at the device level, to enable in-situ recovery from mechanical damages and ensure continuous and stable energy output. Yet, research in this regard remains unexplored.

In this study, we propose a swift modular assembly strategy to fabricate quasi-solid-state TEC devices, particularly applicable to flexible low-grade heat harvest applications. By employing MXene nanosheets as simultaneous accelerators and crosslinkers, hydrogel electrolytes with adhesive surfaces and self-healing capability can be promptly obtained. Utilizing carbon nanotube papers and polyurethane films as electrodes and substrates, respectively, hydrogel-based TEC devices can be readily assembled without the necessity for any binder or solder. The resulting TEC devices exhibited favorable thermoelectrochemical performance at near room temperature, high mechanical flexibility and stretchability to accommodate human body motions, and desired device-level self-healing capability to recover mechanical damages during operation. They could efficiently harvest human body heat and have been successfully applied in encrypted communication and utilized as energy-autonomous sensors to monitor various body movements. The proposed easy and effective assembly strategy of TEC devices offers a promising energy solution for the development of flexible and wearable electronics.

## Experimental Section

### Materials

Acrylic acid (AA), ammonium persulphate (APS), Potassium ferricyanide [K_3_(Fe(CN)_6_)], and potassium ferrocyanide [K_4_Fe(CN)_6_] were purchased from Shanghai Macklin Biochemical Technology Co., Ltd. Guanidine hydrochloride (CH_6_ClN_3_), lithium fluoride (LiF), concentrated hydrochloric acid (HCl), Ti_3_AlC_2_ powder were obtained from Shanghai Aladdin Biochemical Technology Co., Ltd. Carbon nanotube papers (CNTPs) were purchased from Suzhou Hengqiu Nanotech Co., Ltd. All chemicals were used as received without any further purification or treatment. Deionized water was utilized in all experiments.

### Preparation of Ti_3_C_2_T_***x***_ MXene Nanosheets

Ti_3_C_2_T_*x*_ nanosheets were synthesized using a selective etching method, employing LiF/HCl as the etching solution. Briefly, 1 g of Ti_3_AlC_2_ powder was added to 20 mL of 50% HF solution with continuous stirring. The solution was then stirred at 60 °C to ensure even dispersion. After etching, the resultant Ti_3_C_2_T_*x*_ MXene flakes were rinsed with deionized water until the pH reached 6–7, followed by drying in a vacuum oven to obtain the Ti_3_C_2_T_*x*_ powders. Finally, the powders were dispersed in 20 mL deionized water and sonicated in an ice bath for 90 min to produce a homogenous suspension of exfoliated Ti_3_C_2_T_*x*_ MXene nanosheets.

### Preparation of Hydrogels

For the synthesis of pure PAA hydrogel, 3 g of AA monomer was initially dissolved in 4 mL of deionized water, followed by the addition of 3 mg of APS as the initiator. The precursor solution was degassed through ultrasonic treatment and poured into glass molds. The hydrogel formation occurred at room temperature over the course of a week. For PAA-MXene hydrogel preparation, 0.045 mg of MXene nanosheets dissolved in 9 mL of deionized water were added to the PAA precursor solution prior to polymerization. To prepare PAA-MXene/HCF-GdmCl, the previously prepared PAA-MXene hydrogel was immersed in a mixed solution of K_3_Fe(CN)_6_/K_4_Fe(CN)_6_ and CH_6_ClN_3_ at varying concentrations at room temperature for 2 h.

### Morphological and Structural Characterizations

The sample's micromorphology was observed using an atomic force microscope (AFM, Bruker Dimension ICON), a high-resolution scanning electron microscope (SEM, Thermo APREO S) at an acceleration voltage of 5 kV, and a transmission electron microscope (TEM, JEOL F200) at an acceleration voltage of 120 kV. FTIR spectra were captured using a Fourier transform infrared spectroscopy (ATR-FTIR, PerkinElmer Spectrum 3), with a scan in the wavenumber range of 4000–1000 cm^−1^, 64 scans, and a nominal resolution of 4 cm^−1^ at room temperature. The Raman spectra of the hydrogel were collected with a Raman spectrometer (Thermo Fisher DXR XI) using a laser at an excitation wavelength of 532 nm.

### Mechanical Property Tests

The stress–strain curves were obtained using a tensile machine (SANS EUT4103) with a 50 N load at a rate of 50 mm min^−1^ in air under room temperature. The hydrogel samples were set to the dimensions of 3.0 mm (thickness), 30 mm (length), and 10 mm (width), with the gauge length set to 15 mm.

### Thermoelectrochemical Performance Measurements

The *S*_e_ of the TECs was calculated from the slope of the thermoelectrochemical potential (Δ*V*) versus temperature difference (Δ*T*) curves (*S*_e_ = −Δ*V*/Δ*T*), recorded on a custom-built apparatus consisting of a cold plate, a hot plate, a temperature control system, and a Keithley 2700 Data Acquisition (Solon, Ohio, USA). The output voltage (*V*) and output current (*I*) were recorded with varying load resistance. The output power (*P*) was calculated using the formula *P* = *I* × *V*. The ionic conductivity of the TECs was measured using electrochemical impedance spectroscopy on an electrochemical workstation (Chenhua 660e) at room temperature. The ionic conductivity (*σ*) was determined from the Nyquist plot via the equation *σ* = *L*/(*R*_b_ × *A*), where *L* (cm) is the distance between the two electrodes, *R*_b_ (Ω) is the bulk resistance (intercept at the *Z*′ axis), and *A* (cm^2^) is the contact area between the sample and the electrodes.

### Fabrication of the Strip TEC Array and the Self‐Powered Motion Sensor

For the strip TEC array, the hydrogel electrolytes were cut into 1 × 1 cm^2^ squares with CNTPs used as electrodes and connecting wires. The fabricated TECs were then thoroughly wrapped with polyurethane films. For the self-powered motion sensor, the fabricated TECs were equipped with CNTP electrodes at the ends and padded with polyimide tape to prevent short circuiting. One end of the sensor was in direct contact with the skin, while the other was separated from the skin by a heat insulation layer, thus creating a temperature difference. The sensors were affixed to various parts of the human body for motion detection, and the corresponding external circuit voltage and current were recorded using a Keithley 2700 Data Acquisition.

## Results and Discussion

**Fig. 1:**
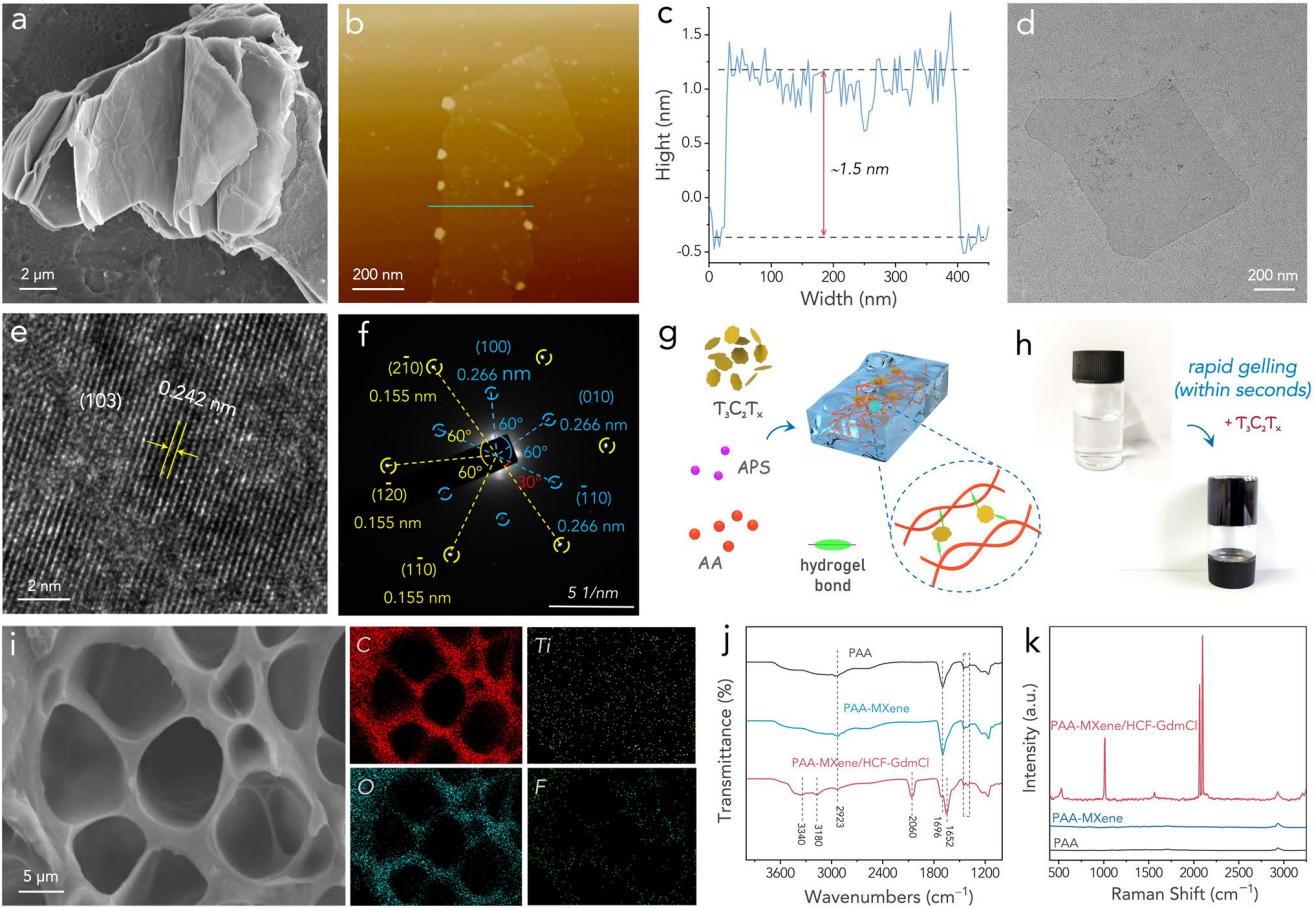
**a** SEM image of the stacked Ti_3_C_2_T_*x*_ MXene nanosheets after etching. **b** AFM image of the exfoliated Ti_3_C_2_T_*x*_ MXene nanosheet. **c** The corresponding height profile. **d** TEM image of the exfoliated Ti_3_C_2_T_*x*_ MXene nanosheet. **e** High-resolution TEM image of the exfoliated Ti_3_C_2_T_*x*_ MXene nanosheet. **f** SAED pattern of the exfoliated Ti_3_C_2_T_*x*_ MXene nanosheet. **g** An illustration of the gelation process of the PAA-MXene hydrogel. **h** Optical images before and after gelling. **i** SEM image of the PAA-MXene hydrogel with corresponding elemental mapping images. **j** FTIR spectra of various samples. **k** Raman spectra of various samples

The typical gelation process of poly(acrylic acid) (PAA) take hours in the presence of ammonium persulfate (APS) initiator at an elevated temperature, which is time consuming and energy-inefficient [39–41]. Ti_3_C_2_T_*x*_ MXene, a hydrophilic two-dimensional transitional metal carbide, can activate rapid gelation of PAA hydrogels within seconds under ambient temperature [42–44], offering a more efficient alternative. The Ti_3_C_2_T_*x*_ MXene nanosheets were synthesized by selectively etching aluminum layers from their parent MAX phase (Fig. 1a). This was followed by the exfoliation of these materials into ultrathin nanosheets, facilitated through a sonication process. To assess the morphological characteristics of the resultant nanosheets, atomic force microscopy (AFM) and transmission electron microscopy (TEM) were utilized. The AFM micrograph (Fig. 1b) distinctly illustrates the shape of a Ti_3_C_2_T_*x*_ flake, complete with well-defined edges. Figure 1c depicts the flake thickness to be approximately 1.5 nm, indicative of the successful synthesis of monolayer MXene flakes. The exceptionally thin structure and sharp edges of the Ti_3_C_2_T_*x*_ are further corroborated by the TEM image presented in Fig. 1d. The thin monolayer Ti_3_C_2_T_*x*_ preserves good crystallinity, as shown in the high-resolution TEM image in Fig. 1e, where an inter-planar spacing of 0.242 nm corresponding to the (103) plane of Ti_3_C_2_T_*x*_ can be identified [45]. The corresponding selected area electron diffraction (SAED) pattern in Fig. 1f further reveals the single crystallinity with hexagonal symmetry of the exfoliated Ti_3_C_2_T_*x*_ flake, where the {100} and {110} planes of Ti_3_C_2_T_*x*_ are evidently observed [46]. This comprehensive analysis affirms the successful synthesis and notable morphological characteristics of the Ti_3_C_2_T_*x*_ nanosheets.

The PAA-MXene hydrogels were synthesized through in situ free-radical polymerization, as illustrated in Fig. 1g, where APS and Ti_3_C_2_T_*x*_ nanosheets were employed as the initiator and crosslinker, respectively. Remarkably, gelation occurred within seconds at ambient conditions, eliminating the need for heat or ultraviolet light facilitation (Fig. 1h). Conversely, a precursor devoid of MXene only yielded a weak monolith or partial hydrogel after two days (Fig. S1). This rapid and instantaneous gelation process, stimulated by Ti_3_C_2_T_*x*_ MXene, can be credited to the cumulative self-heating effect arising from several factors [44, 47, 48]: (1) The pronounced exothermic reaction initiated within seconds due to the potent chelation between the carboxyl groups of acrylic acid monomers and the surface functional groups of Ti_3_C_2_T_*x*_ nanosheets. The low thermal conductivity of the aqueous precursor solution restricted the dissipation of the resultant heat. (2) Following the onset of polymerization, the incorporation of MXene nanosheets prompted substantial heat release during polymerization. This effect further accelerated the generation of free radicals, thereby facilitating more rapid crosslinking. (3) Concurrently, a multitude of hydrogen bonds, acting as physical crosslinks, formed between the carboxyl group of the PAA chain and the polarized end group of the Ti_3_C_2_T_*x*_ MXene nanosheets. This bonding resulted in additional intense heat release, thereby enhancing the self-healing properties of the hydrogels through the formation of numerous hydrogen bonds. Although some studies have reported the superiority of MXene in various hydrogel polymerizations [44, 49], these research efforts did not focus on three main aspects that our study specifically addresses: (1) The exploitation of body heat for energy-autonomous wearable applications, (2) The development of self-healability at a device level, and (3) The quick assembly of wearable devices.

Scanning electron microscopy (SEM) image in Figs. 1i and S2 reveal the porous structure of the hydrogel. This porosity ensured efficient mass transport during the thermoelectrochemical process when utilized as a TEC electrolyte, a characteristic vital for redox ion replenishment and the progression of redox reactions at the electrolyte–electrode interface [50, 51]. Elemental mapping images presented in Fig. 1i show the homogeneous distribution of Ti and F elements, suggesting the well dispersion of the Ti_3_C_2_T_*x*_ nanosheets in PAA matrix. After gelation, the Fe(CN)_6_^3−/4−^ hexacyanoferrates (HCF) redox ions and guanidinium chloride (GdmCl) were incorporated through ion exchange, affording a hydrogel electrolyte that was subsequently responsible for the high thermopower of the TEC (discussed later). The structures of the pristine PAA hydrogel, PAA-MXene hydrogel, and the PAA-MXene/HCF-GdmCl hydrogel electrolyte were examined using Fourier-transform infrared spectroscopy (FTIR). As shown in Fig. 1j, the peaks at 1695 and 1400–1500 cm^−1^ were identified as the anti-symmetric and symmetric stretching vibrations of carboxylic acid groups in PAA chains, respectively [40, 52]. The addition of HCF and GdmCl resulted in new peaks emerged at 2060 and 1652 cm^−1^, associated with the vibrations of C≡C and C=N groups, respectively [24]. Raman spectra (Fig. 1k) reveal the characteristics bands of PAA at 2936, 1709, 1458, 1104 and 850 cm^−1^, attributed to C–H stretching, C=O stretching, –CH_2_ bending, –OH in-plane bending and C–COOH stretching vibrations, respectively [44, 53]. Additionally, the PAA-MXene/HCF-GdmCl hydrogel electrolyte exhibited two sharp peaks at 2098 and 2063 cm^−1^, corresponding, respectively, to the A_1g_ and *E*_g_ modes of C≡N in GdmCl molecules [54, 55]. An intense peak at 1015 cm^−1^ was also observed, which is ascribed to the symmetric N–C–N stretching vibration [56]. X-ray photoelectron spectroscopy (XPS) spectra in Figs. S3 and S4 further confirm the successful incorporation of Ti_3_C_2_T_*x*_ MXene as well as HCF and GdmCl in the hydrogel electrolyte.

**Fig. 2:**
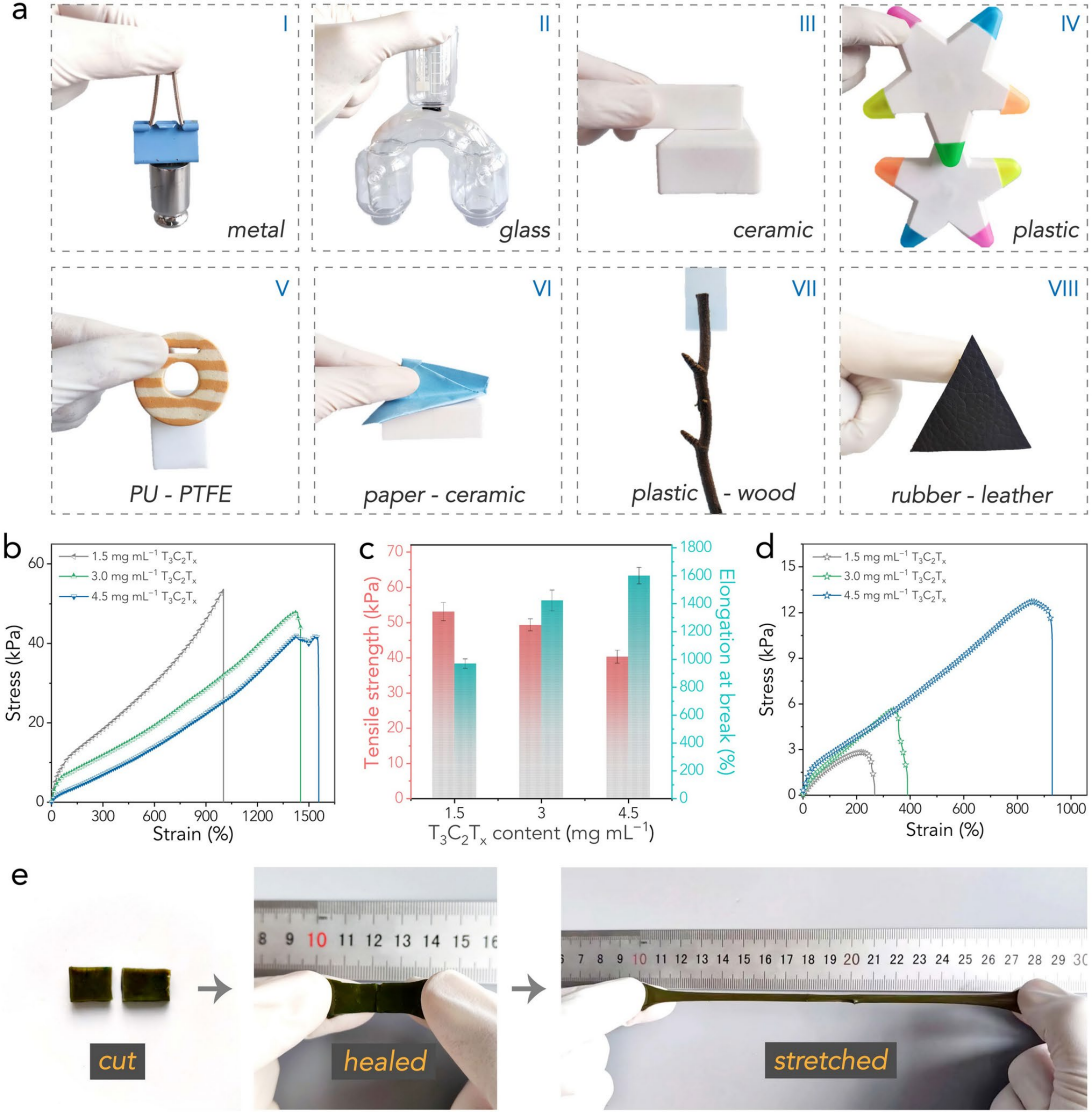
**a** Optical images showing the high adhesiveness of the hydrogel electrolyte to various surfaces. **b** Stress–strain curves of various samples. **c** Tensile strength and elongation at break of various samples. **d** Stress–strain curves of various samples after self-healing. **e** Optical images demonstrating the stretching process of the hydrogel electrolyte after self-healing

Combining the carboxyl groups on PAA chains with the abundant surface functional groups on Ti_3_C_2_T_*x*_ nanosheets, the introduction of MXene into the hydrogels promoted exceptional adhesion to a wide range of surfaces, both organic and inorganic. This characteristic negates the necessity for additional adhesive tapes and straps for subsequent device assembly and long-term operation [44, 57]. This capability is demonstrated in Fig. 2a. As shown, two metallic objects were glued together by the PAA-MXene/HCF-GdmCl hydrogel, which could sustain a weight of 200 g. Note that the hydrogel electrolyte firmly adhered to the metal surface without additional adhesives. It also manifested strong adhesion to glass, ceramic, and plastic surfaces. Moreover, different surfaces such as plastic foam (polyurethane, PU)-dense plastic (polytetrafluoroethylene, PTFE), paper-ceramic, plastic-wood, and rubber-leather could be intimately bound together using the hydrogel electrolyte. The excellent and versatile adhesiveness lays a good foundation for the swift device assembly, eliminating the requirement for any binder or solder.

The mechanical properties of various hydrogel electrolytes were evaluated by tensile tests. As revealed by the stress–strain results in Fig. 2b, c, the incorporation of MXene led to enhanced stretchability, proving the efficacy of the 2D Ti_3_C_2_T_*x*_ nanosheets as effective crosslinkers [40]. Notably, the hydrogel electrolyte prepared with 4.5 mg mL^−1^ MXene exhibited considerable stretchability, exceeding 1500% elongation while maintaining an elastic modulus of 41.9 kPa (the stretchability and strength were demonstrated in Figs. S5 and S6). Given that Ti_3_C_2_T_*x*_ nanosheets naturally possess abundant oxygen-containing surface groups, they can form multiple hydrogen bonds with PAA chains, thereby guaranteeing exceptional self-healing capability via reversable physical crosslinking. Specifically, PAA-MXene-1.5 (where 1.5 denotes the MXene content in mg mL^−1^) sustained a healed strain of 221%; remarkably, the healed strains for PAA-MXene-3.0 and PAA-MXene-4.5 reached 350% and 855%, respectively, after a 6-h healing period (Fig. 2d). Further raising the MXene concentration led to even faster gelation, but this resulted in an inhomogeneous hydrogel electrolyte and reduced the mechanical strength of the hydrogel electrolytes. Therefore, the maximum MXene concentration was set at 4.5 mg mL^−1^. The efficient self-healing capability of PAA-MXene-4.5 was demonstrated in Fig. 2e and Video S1: a hydrogel electrolyte, once cut into two halves, can be easily recovered through a 6-h self-healing process. The healed sample can be manually stretched to several times its original length without breakage, and the wound after healing was shown in Fig. S7. Despite these manipulations, the hydrogel electrolyte retained its robust adhesion effect, as shown in Fig. S8, where it was used to hang a plastic object without any binder.

The hydrogel electrolyte, containing HCF redox ions, can generate a potential difference between two electrodes when exposed to a temperature gradient, thus realizing heat-to-electricity conversion. This feature is especially relevant for wearable applications that demand stretchability and self-healing capabilities. To evaluate these attributes, we thoroughly investigated the thermopower of the hydrogel electrolyte under conditions of large stretching strains, repeated stretching, sustained stretching, and multiple self-healing cycles.

**Fig. 3:**
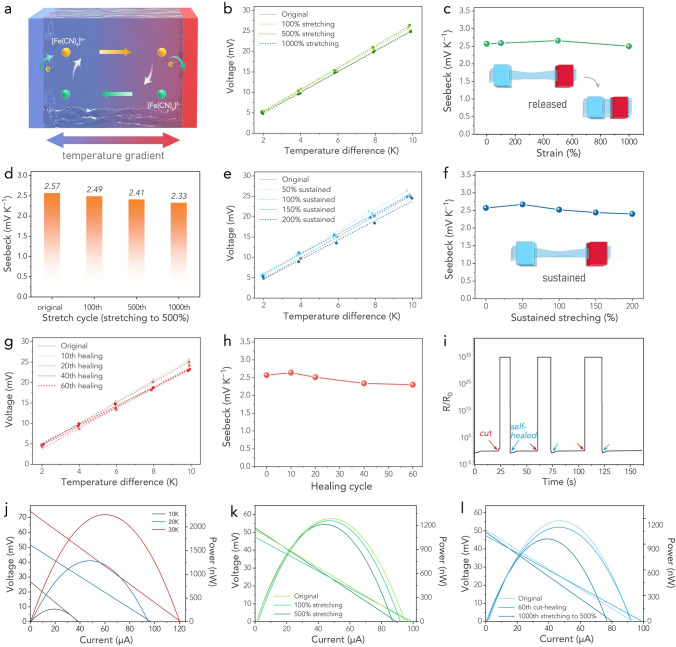
**a** An illustration of the working mechanism of the TEC. **b** The generated voltage as a function of temperature difference after being stretched. **c** Measured *S*_e_ values after being stretched. **d** The measured *S*_e_ values after multiple stretching cycles at 500% strain. **e** Generated voltage as a function of temperature difference under sustained stretching. **f** Measured *S*_e_ values under sustained stretching. **g** Generated voltage as a function of temperature difference after multiple self-healing cycles. **h** Measured *S*_e_ values after multiple self-healing cycles. **i** The resistance variations after multiple self-healing cycles. Voltage, current, and power output of **j** the TEC under the temperature difference of 10 K, 20 K, and 30 K, **k** the TEC after being stretched under the temperature difference of 20 K, **l** the TEC after multiple stretching and cut-healing cycles under the temperature difference of 20 K

Prior to that, the influence of GdmCl and HCF contents on thermopower was examined. As illustrated in Fig. 3a, in a typical TEC, a redox couple-containing electrolyte is placed between two electrodes, and these two electrodes are exposed to different temperatures, affording a temperature gradient across the TEC body. The temperature dependence of redox potentials creates a potential difference across the cell via redox reactions, and the corresponding *S*_e_ can reach the order of millivolts per Kelvin. Specifically, during a typical thermoelectrochemical process, [Fe(CN)_6_]^4−^ at the hot anode releases an electron and oxidizes to [Fe(CN)_6_]^3−^. In contrast, at the cold cathode, [Fe(CN)_6_]^3−^ accepts an electron and reduces back to [Fe(CN)_6_]^4−^. This sequence of events generates a potential difference between the anode and the cathode, subsequently triggering a consistent flow of electrons in the external circuit. The generated thermopower was determined from the open-circuit voltages (OCVs) over a range of temperature differences (Δ*Ts*). As shown in Fig. S9, the OCVs were linearly proportional to the inter-electrode Δ*Ts*. The slope of the fitting curve determines the *S*_e_, as defined by the expression *S*_e_ = *∂V*/*∂T* = Δ*S*_*B,A*_/*nF*, where *T* is the temperature, *V* is generated potential, *n* is the number of electrons transferred in the redox reaction, *F* is the Faraday constant, and Δ*S*_*B,A*_ is the reaction entropy for the involved redox couple [58, 59]. GdmCl was introduced into the hydrogel electrolyte in pursuit of large thermopower; guanidinium is prone to bond with [Fe(CN)_6_]^4−^ rather than [Fe(CN)_6_]^3−^ based on the ion specificity, and this difference in affinity can enlarge the entropy difference of the redox couple, thereby amplifying the thermoelectrochemical Seebeck effect [25]. As shown in Fig. S10, the hydrogel electrolyte without GdmCl showed a typical thermoelectrochemical Seebeck coefficient *S*_e_ of ~ 1.4 mV K^−1^, and this value reached a peak of 2.57 mV K^−1^ at a GdmCl concentration of 2.5 mol L^−1^, indicating the effective enhancement in thermopower by adding GdmCl. Meanwhile, as we initially increased the GdmCl concentration, ion transport within the hydrogel electrolyte network was facilitated. However, when the GdmCl concentration was further increased, a notable decrease in the ionic conductivity was observed. This effect is likely attributable to the hindrance of effective ionization and conduction of the redox ions due to excessively high GdmCl concentrations [32]. As a result, the ionic conductivity peaked at the GdmCl concentration of 2.5 mol L^−1^ (Fig. S11). Using the formula of power factor PF = *S*^2^*σ*, we subsequently acquired a maximum PF value of 30.94 mW m^−1^ K^−2^ at a GdmCl concentration of 2.5 mol L^−1^ (Fig. S12), suggesting that at this concentration, the hydrogel electrolyte could perform best regarding both thermopower and mass transport.

The *S*_e_ fitting plots for the hydrogel, stretched to 100%, 500%, and 1000% strain, are presented in Fig. 3b. The corresponding variation in values is depicted in Fig. 3c. The *S*_e_ values are generally in the range of 2.50–2.70 mV K^−1^, suggesting that even a 1000% stretch only marginally impacts thermopower, causing less than a 10% fluctuation. The thermopower under various sustained stretching states were also measured, as shown in Fig. 3d, e. The *S*_e_ values showed less than 10% fluctuations at either 50%, 100% or 150% stretching states compared to the original state, suggesting that the thermoelectrochemical reactions could successfully occur under large deformations. Figure 3f further compares the thermopowers after repeated stretching. The results show that the hydrogel electrolyte can sustain a *S*_e_ of 2.33 mV K^−1^ even after 1000 cycles of 500% stretching. In addition to stretching, the impact of self-healing on thermopower was also investigated. The hydrogel underwent multiple self-healing, and the corresponding *S*_e_ values gradually decreased with increasing self-healing times. However, even after 60 healing cycles, the *S*_e_ remained at approximately 2.30 mV K^−1^, corresponding to ~ 90% of the initial value prior to any healing (Fig. 3g, h). Moreover, the hydrogel rapidly regained its conductivity once it was self-healed, suggesting promising potential for sensing applications (Fig. 3i). The stable thermopower under conditions of substantial, repeated, and sustained deformations, as well as through repeated cut-healing cycles, ensures a reliable energy output for subsequent wearable applications, making the hydrogel electrolyte a promising candidate for integration into wearable technology, capable of transforming thermal energy into electric power under various operating conditions.

The performance output of the TEC using the as-fabricated hydrogel electrolyte and platinum electrodes was evaluated at different temperature gradients (Δ*Ts*), as presented in Fig. 3j. Under the Δ*T* of 10, 20, and 30 K, the short-circuit current was observed to be 38.8, 97.3, and 121.2 µA, respectively. Correspondingly, the maximum output power (*P*_max_) was 267.9, 1286.5, and 2258.5 nW. The TEC maintained *P*_max_ at 1260.3 and 1215.2 nW under the Δ*T* of 20 K, even after being stretched by 100% and 500%, respectively (Fig. 3k). Remarkably, the TEC also retained a *P*_max_ of 1032.1 nW under the Δ*T* of 20 K, even when stretched to 500% for 1000 cycles (Fig. 3l), corresponding to 80% of its initial state. Most impressively, despite undergoing a cut-healing process for 60 cycles, the TEC was able to sustain a *P*_max_ of 1179.1 nW, approximately 92% of its initial state. These findings firmly attest to the robust thermoelectrochemical performance of the TEC, even under extensive deformations and repeated damage-healing cycles.

**Fig. 4:**
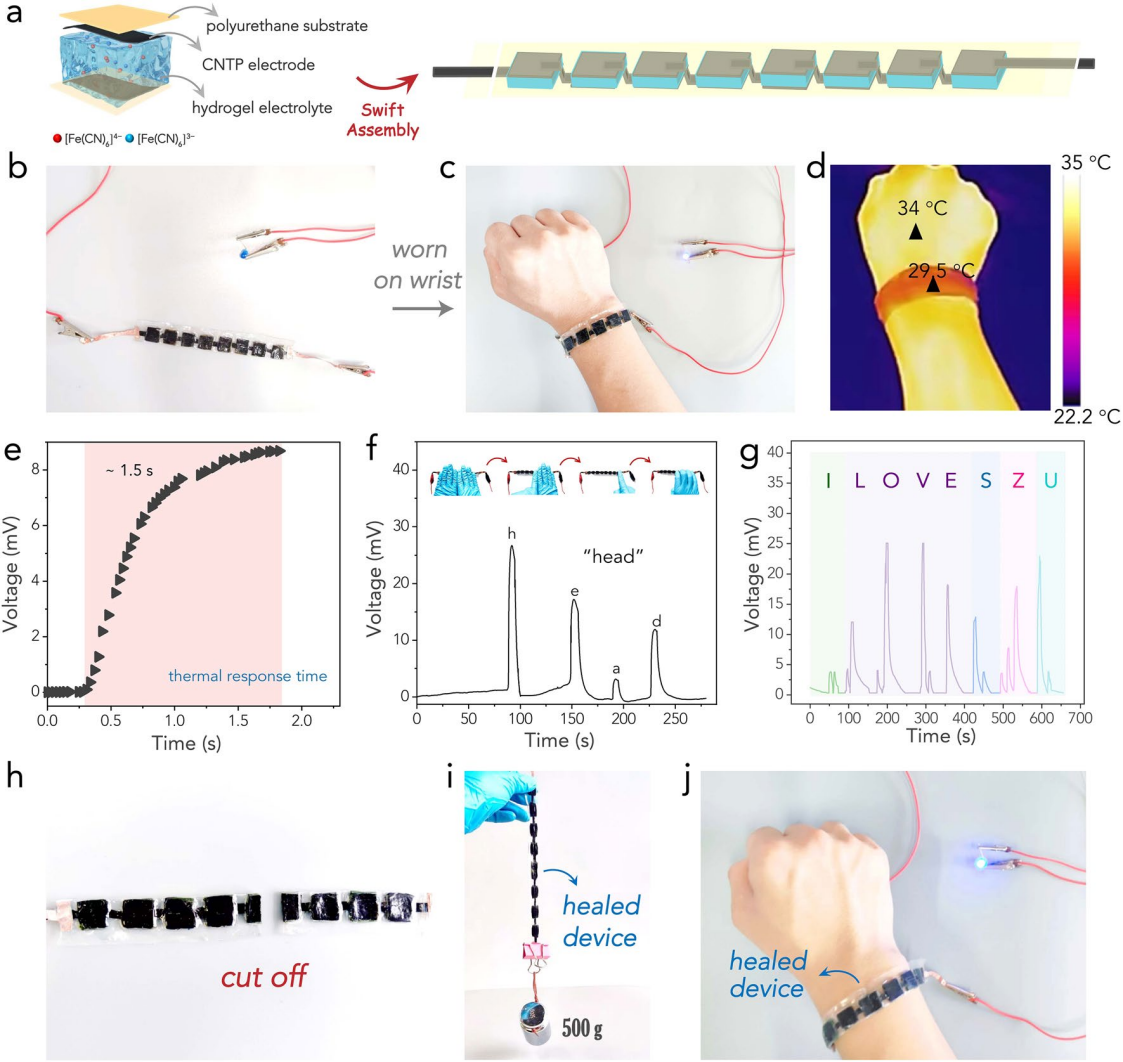
**a** Illustrations of the structure of a single TEC and the as-assemble strip TEC array. **b** An optical image showing the strip device connected to an LED bulb. **c** An optical image showing the strip device worn on wrist to power an LED bulb (with the assistance of a voltage amplifier). **d** Corresponding thermal image. **e** Fast voltage response to heat of the device. **f** A demonstration of using the voltage signal to encrypt the word “head”. **g** A demonstration of using the voltage signal to encrypt the phrase “I LOVE SZU”. **h** An optical image showing the strip device cut into two halves. **i** An optical image showing the self-healed strip device to support a 500 g weight. **j** An optical image showing the self-healed strip device to power an LED bulb by harvesting body heat

Owing to its high adhesion, good flexibility/stretchability, and attractive self-healing capability, the PAA-MXene hydrogel electrolyte was seamlessly incorporated into a TEC array device within minutes, eliminating the need for any binder or solder. In this assembly, the hydrogel electrolyte was cut into small sections, approximately 10 mm × 10 mm × 5 mm in size. Carbon nanotube papers (CNTPs) served as both electrodes and connecting circuits, and eight individual TECs were connected in series to form an array. Thin polyurethane films acted as substrates on both sides to create an integrated strip device (Fig. 4a, b). This device demonstrated excellent adaptability and could be comfortably worn on the wrist (Figs. 4c and S13). Harnessing body heat, this device successfully powered a light-emitting diode (LED) bulb with the aid of a voltage amplifier, as demonstrated in Video S2. Infrared thermal imaging (Fig. 4d) showed a body temperature of 34.0 °C, while the device's surface temperature facing the air was 29.5 °C, thus creating an approximate temperature difference of 4.5 °C between the device and the wrist. Note that the actual utilized temperature was much lower, as the polyurethane substrates and CNTP electrodes inevitably inhibited the heat conduction to electrolyte. Figure 4e indicates that the device responded swiftly to low-grade heat, generating an output voltage of ~ 9 mV in less than two seconds.

The strip device, consisting of eight TECs, effectively harvested body heat, enabling the generation of different voltage signals by altering finger-touching behaviors. For instance, touching all eight TECs with eight fingers produced a ~ 27 mV voltage signal, while touching one TEC with one finger resulted in a ~ 3.3 mV signal. This mechanism allowed for the transmission of distinct signals through different touch actions. Each letter of the alphabet could be represented by a unique voltage signal, enabling the device to encrypt words and phrases through controlled touch behaviors. For demonstration, the word "head" and the phrase "I LOVE SZU" were successfully encrypted into voltage signals by the device in real-time, taking advantage of its rapid heat response time (Fig. 4f, g). The codes for encrypting all 26 alphabetic letters via voltage signals are provided in Fig. S14. To encrypt a broader range of information, the device could be redesigned with smaller TEC pieces in a more suitable arrangement way, so that one finger touch can cover more TEC surfaces, generating higher voltage signals. By also incorporating the dimension of time, such as touching for a certain period, voltage signals with varied durations could be created, thereby enhancing the encryption capability of the device. Thus, the TEC array has paved the way for innovative touch-based communication systems and data transmission possibilities.

Leveraging the self-healing attributes of both the hydrogel electrolytes and the polyurethane substrates, the strip device demonstrated robust self-healing at the device level. This was evidenced by directly cutting the device in half (Fig. 4h), and subsequently restoring it with the presence of ethanol for 10 min. The healed device not only regained its physical strength, capable of supporting a 500 g weight without breaking (Fig. 4i), but also retained its ability to harvest low-grade heat. This was demonstrated by reusing it to power an LED bulb via utilizing body heat, even after healing (Fig. 4j). These results unveil the significant potential of the device in wearable applications.

**Fig. 5:**
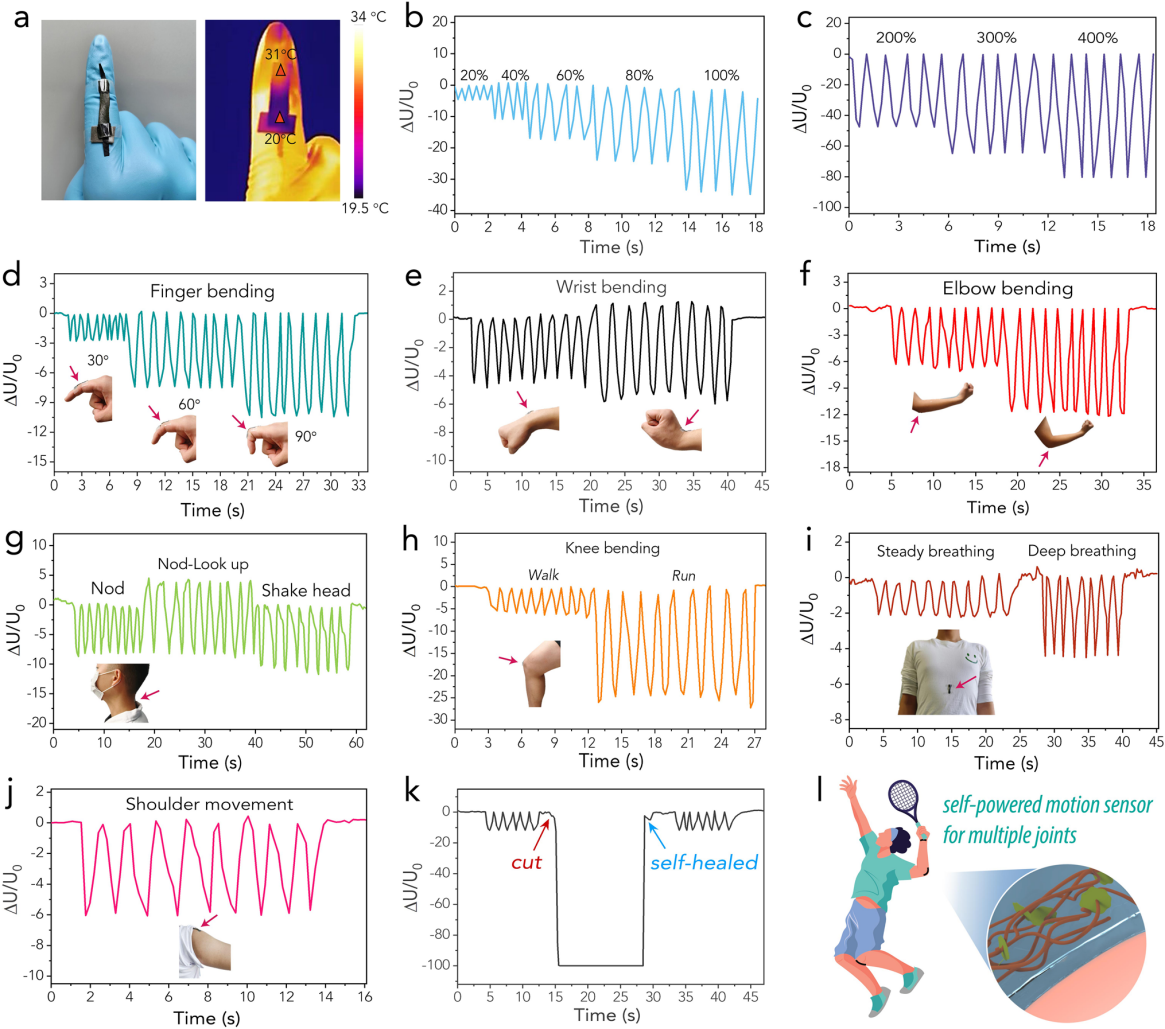
**a** Optical and thermal images of the TEC device fixed onto a finger. **b**, **c** Time-dependent voltage variation of the TEC under various tensile strains. **d**–**j** Voltage variation of the self-powered sensor under various body motions, including finger bending, wrist bending, elbow bending, head movements, knee bending, breathing, and shoulder movements. **k** Voltage variation of the self-powered sensor before and after a cut-healing process. **l** An illustration showing using the self-powered sensor to detect complex body movements

Given its high thermoelectrochemical performance and mechanical adaptability, the TEC, underpinned by the PAA-MXene hydrogel, can be readily employed as a self-powered sensor for strain detection. This capability stems from the TEC's ability to generate strain-dependent electrical signals derived from its inherent thermogalvanic effect. As demonstrated in Fig. 5a, the TEC could be conveniently fixed onto a finger, with a heat insulating layer establishing the cold end, thereby creating an effective temperature gradient across the TEC body. The temperature difference between the two ends of the TEC was approximately 11 °C. A voltmeter and an ammeter were used to monitor the voltage and current, respectively, generated by the TEC from the external load in real-time (as illustrated by the circuit in Fig. S15). The variation in voltage (Δ*U*/*U*_0_) and current (Δ*I*/*I*_0_) with strain was recorded (Figs. 5b, c and S17a, b). The thermopower generated at a given temperature difference remained relatively stable within a controlled strain range, while the internal resistance of the TEC increased with the application of greater strain, leading to a decrease in the voltage generated on the load resistor. These electrical signal changes were highly repeatable in the strain range of 20%–400%, confirming the excellent sensitivity of the designed energy-autonomous strain sensor. Gauge factor is a critical metric that quantifies the sensitivity of strain sensors. The relationship between the relative change in resistance (Δ*R*/*R*_0_) and strain (*ε*) is presented in Fig. S16. As can be seen from this figure, the GF values for the PAA-MXene/HCF-GdmCl hydrogels were determined to be 0.88 and 1.08, corresponding to the strain ranges of 0–40% and 40–100%, respectively.

The TEC's high flexibility, excellent strain sensitivity, and stable thermoelectrochemical performance make it particularly suitable for adaptation to various parts of the human body for in-situ monitoring of body movements, including finger bending, wrist bending, elbow bending, head movement, knee bending, breathing, and shoulder movement, as displayed in Figs. 5d–j and S17c–i. For instance, as the bending angle of the finger increased from 30° to 60° and further to 90°, the TEC was correspondingly deformed, resulting in an increase in internal resistance and a decrease in output voltage (consistent with Fig. 5b, c). The TEC exhibited stable and repeatable voltage signals across all motions, from subtle breathing vibrations to large joint movements. This validated the feasibility of using the TEC as a strain sensor powered by efficient low-grade heat harvesting, utilizing the irregularly shaped skin surface as the heat source. In addition, the effect of the self-healing behavior on sensing capability was evaluated. As shown in Figs. 5k and S18, the TEC continued to produce stable and repeatable voltage signals upon finger bending, and these signals remained highly reproducible after undergoing a cut-healing process, signifying the effective recovery of the TEC's strain sensing capability via self-healing. The successful application of the TEC for energy-autonomous strain sensing represents a significant step toward the vision of employing energy-autonomous wearable electronics to capture complex human actions comprising various body movements (Fig. 5l). Compared to the widely studied triboelectric nanogenerators (TENGs) and piezoelectric nanogenerators (PENGs) for energy-autonomous applications, the key advantage of TECs is their ability to utilize low-grade heat, which is a widely available and often-wasted resource. Unlike TENGs and PENGs, which depend on mechanical stress or strain [60, 61], TECs can continuously harvest thermal energy from the human body or ambient sources without the requirement of any mechanical movement, providing a stable power source.

## Conclusions

In summary, this study has successfully demonstrated a swift assembly approach for flexible TEC arrays. The TEC was constructed on the unique MXene-boosted PAA hydrogel electrolyte, showing excellent energy-harvesting capability, with stable energy output under various challenging conditions, including large, repeated, and sustained deformations, and multiple cut-healing cycles. The resulting hydrogel-based TEC generated a maximum power output of 1032.1 nW under the Δ*T* of 20 K, when subjecting to stretching at 500% for 1000 cycles, corresponding to 80% of its initial state; meanwhile, it sustained a maximum power output of 1179.1 nW under the Δ*T* of 20 K even after 60 cut-healing cycles, approximately 92% of its initial state. More importantly, the hydrogel electrolyte's rapid gelling feature, high flexibility, and good adhesiveness facilitated the swift assembly of a TEC array device, thus overcoming the typical constraint of the complicated fabrication of wearable electronic devices. The resulting TEC array exhibited device-level self-healing capability and high adaptability to human body. It held a fast response to low-grade body heat (within 1.5 s), generating varying voltage signals based on finger-touch behaviors, thus paving the way for innovative touch-based encrypting communication. It was also adapted to various parts of the human body to in-situ monitor a variety of body motions ranging from subtle breathing vibrations to large joint movements, capable of capturing, interpreting, and responding to complex human actions. The prospect of combining these adaptable TEC arrays with other wearable technologies can further advance self-powered wearable electronics, offering a wealth of opportunities for personalized healthcare, fitness monitoring, and human–machine interface systems.

### Supplementary Information

Below is the link to the electronic supplementary material.

Supplementary file1 (PDF 933 kb)

Supplementary file2 (MP4 19042 kb)

Supplementary file3 (MP4 3012 kb)
