# Supplementary material for: Swift Assembly of Adaptive Thermocell Arrays for Device-Level Healable and Energy-Autonomous Motion Sensors
Source: Nanomicro Lett. 2023 Aug 11;15:196. doi: 10.1007/s40820-023-01170-x (PMC10421839; doi:10.1007/s40820-023-01170-x)
Supplement: Supplementary file 1 — Supplementary file1 (PDF 933 kb) [file 40820_2023_1170_MOESM1_ESM.pdf]

Supporting Information for

Swift Assembly of Adaptive Thermocell Arrays for Device-Level Healable and Energy-Autonomous Motion Sensors

Xin Lu[1], Daibin Xie[1], Kaihua Zhu[1], Shouhao Wei[1], Ziwei Mo[1], Chunyu Du[1], Lirong Liang[1], Guangming Chen[1,*] and Zhuoxin Liu[1,2,*]

[1]College of Materials Science and Engineering, Shenzhen University, Shenzhen 518055, P. R. China

[2]State Key Laboratory of Polymer Materials Engineering, Sichuan University, Chengdu 610065, P. R. China

*Corresponding authors. Email: chengm@szu.edu.cn (Guangming Chen); liuzhuoxin@szu.edu.cn (Zhuoxin Liu)

Supplementary Figures

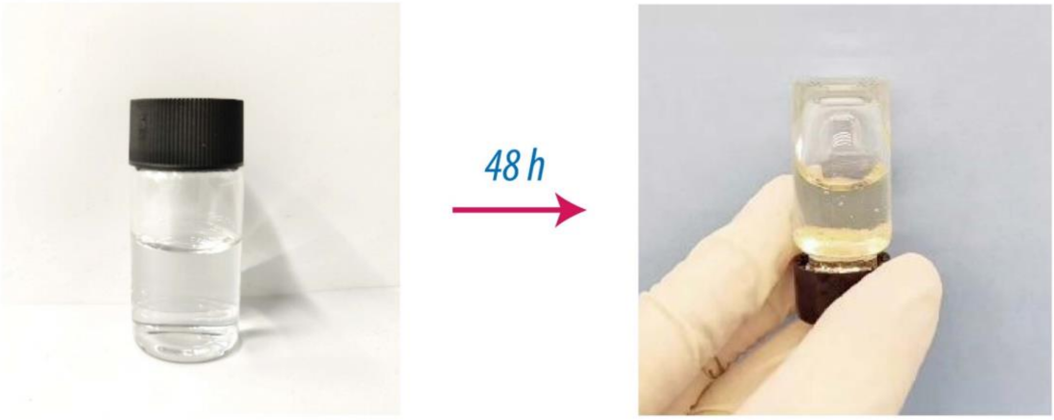

Fig. S1 Optical images of the pure PAA gelation process without the presence of MXene nanosheets

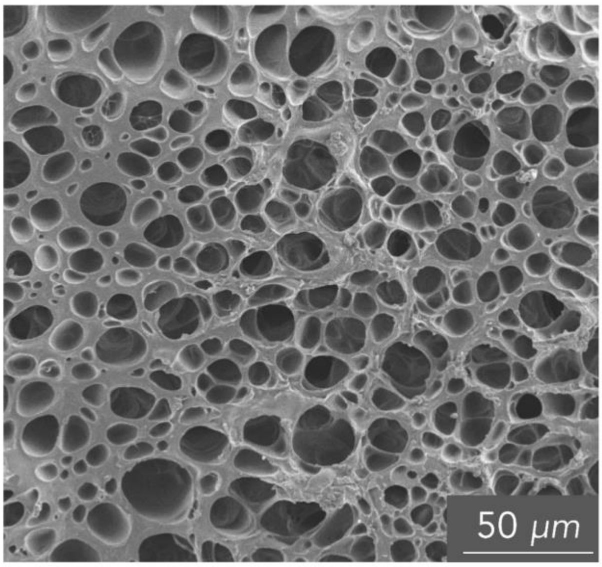

Fig. S2 SEM image of the PAA-MXene hydrogel at a lower magnification

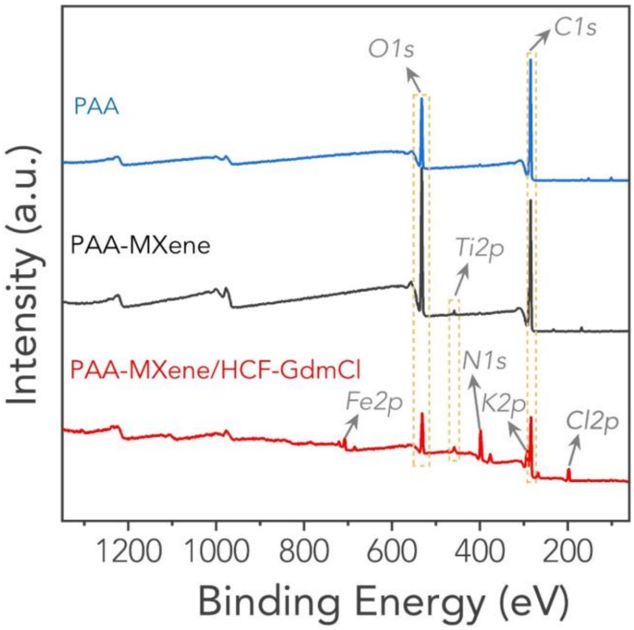

Fig. S3 XPS full spectra of the pure PAA, PAA-MXene, and PAA-MXene/HCF-GdmCl hydrogels

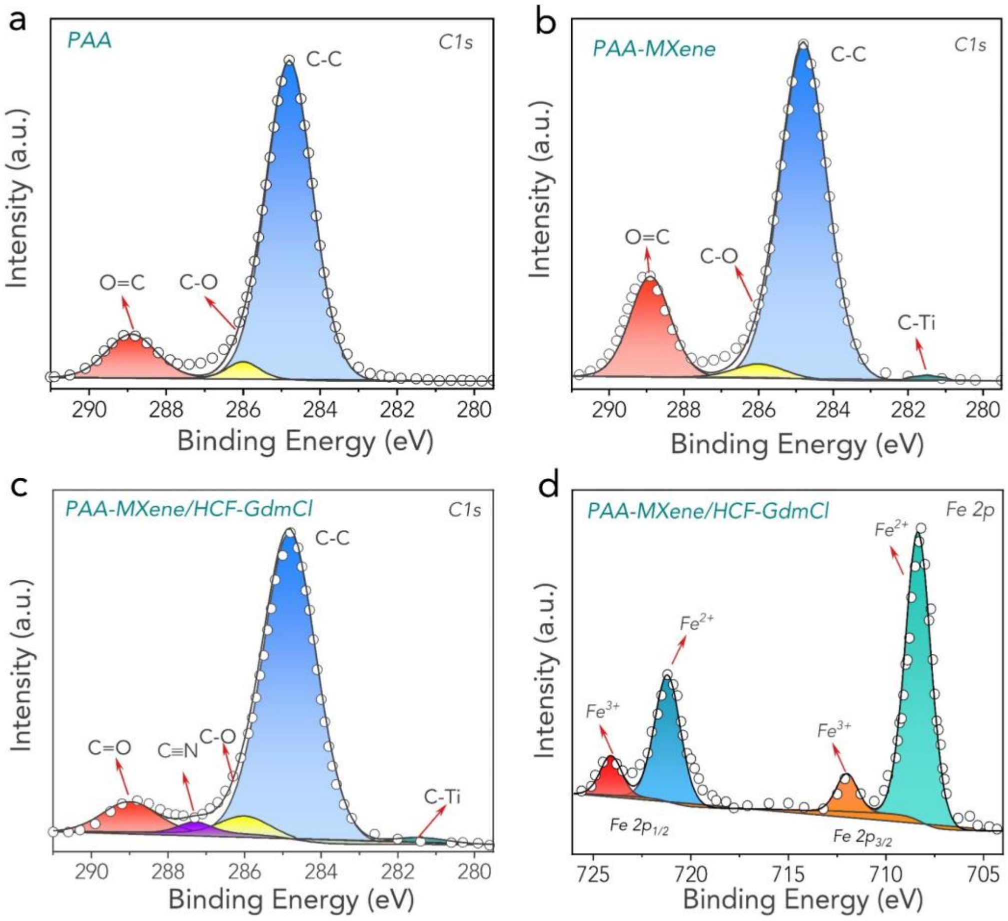

Fig. S4 (**a-c**) XPS C1s spectra of the pure PAA, PAA-MXene, and PAA-MXene/HCF-GdmCl hydrogels. (**d**) XPS Fe2p spectrum of the PAA-MXene/HCF-GdmCl hydrogel

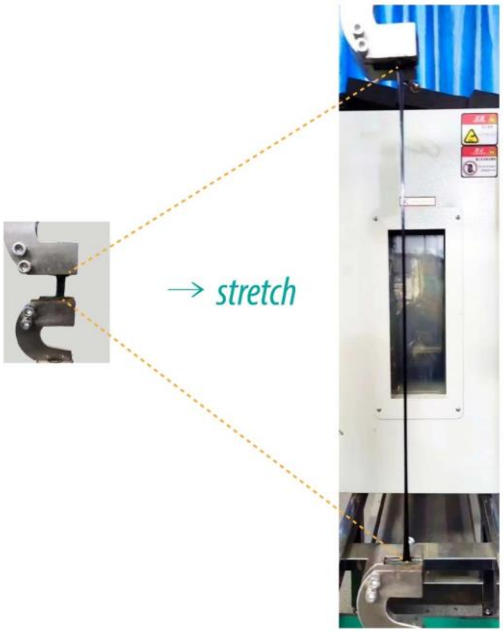

Fig. S5 Optical images showing the high stretchability of the PAA-MXene/HCF-GdmCl hydrogel

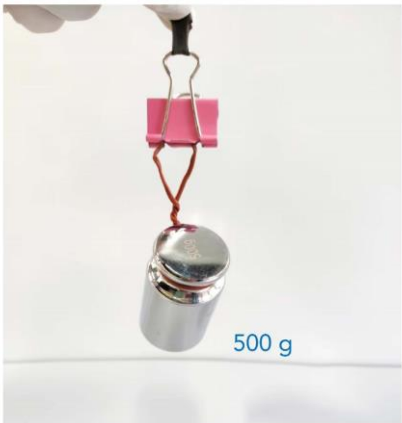

Fig. S6 An optical image showing using the PAA-MXene/HCF-GdmCl hydrogel to hang a 500 g weight

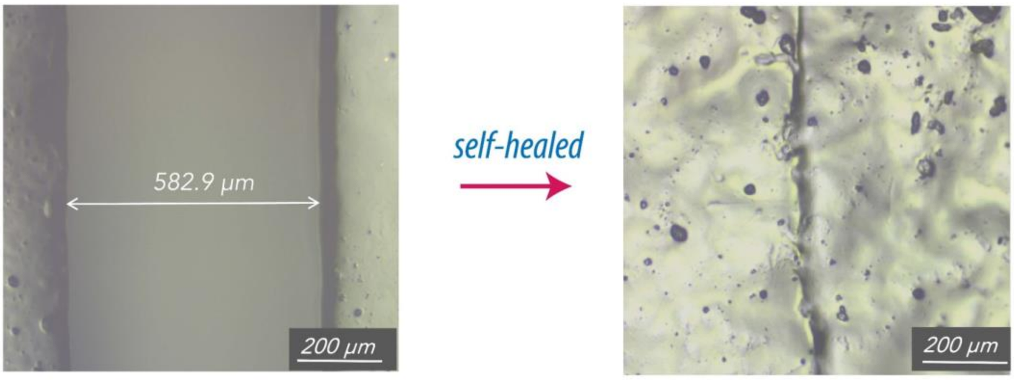

Fig. S7 Microscope images showing the healing wound of the PAA-MXene/HCF-GdmCl hydrogel after cut

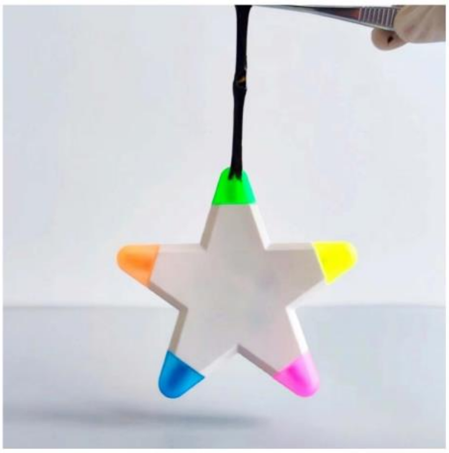

Fig. S8 An optical image showing using the PAA-MXene/HCF-GdmCl hydrogel to hang a plastic object after self-healing

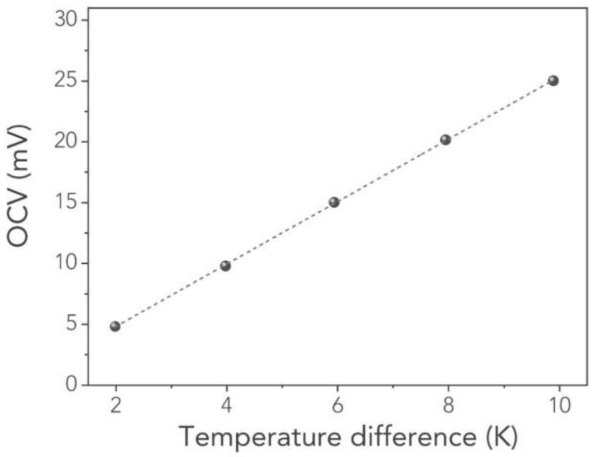

Fig. S9 The generated OCVs as a function of temperature difference by the TEC

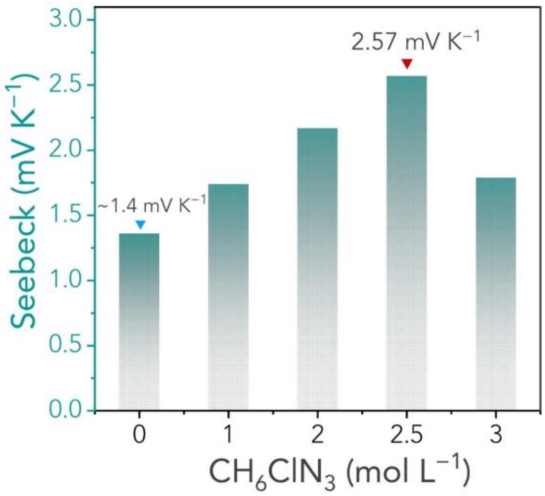

Fig. S10 The effect of CH_6ClN_3 concentration on the S_e of the hydrogel electrolyte

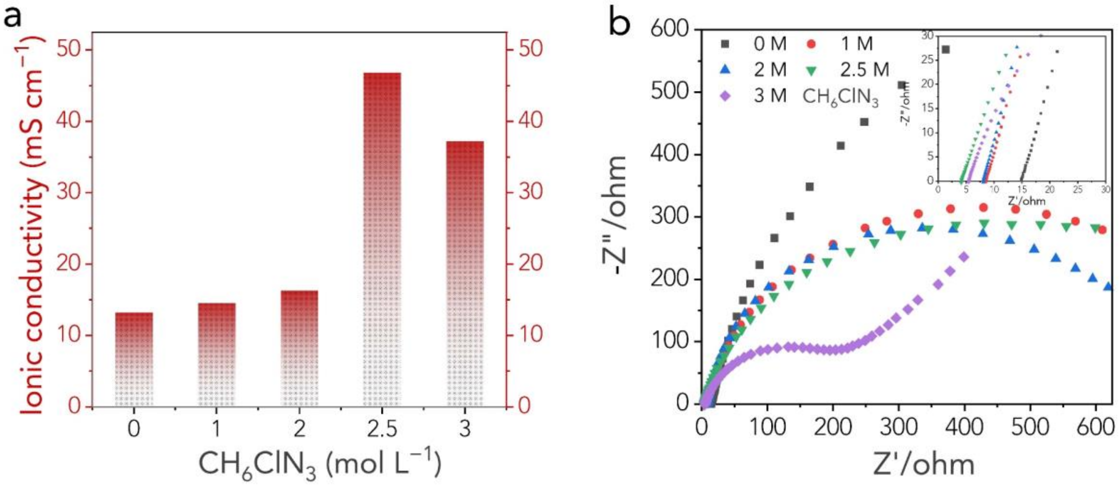

Fig. S11 (**a**) The effect of CH_6ClN_3 concentration on the ionic conductivity of the hydrogel electrolyte. (**b**) The corresponding electrochemical impedance spectra

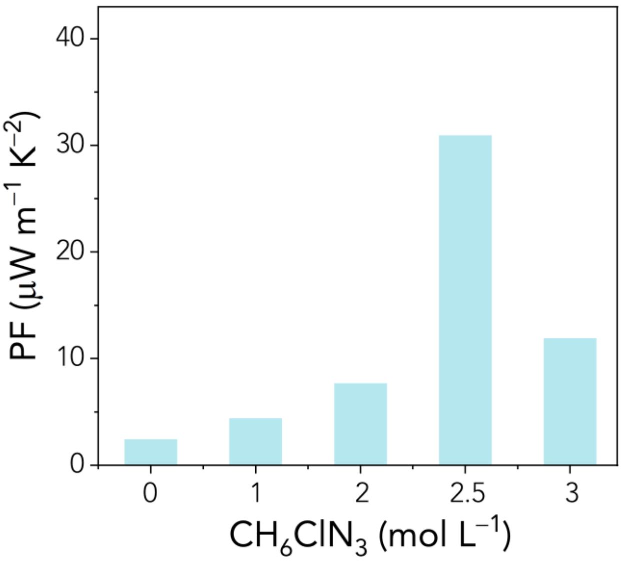

Fig. S12 The effect of CH_6ClN_3 concentration on the *PF* of the hydrogel electrolyte

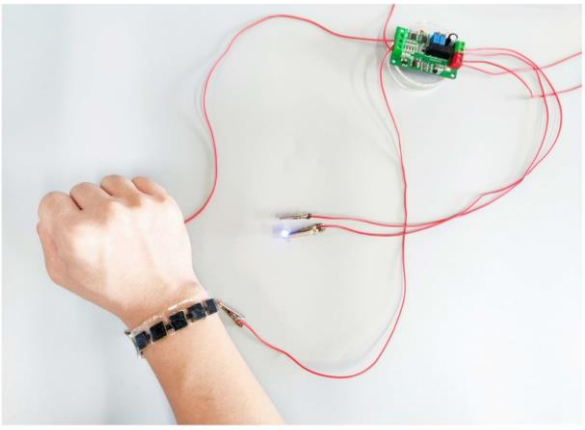

Fig. S13 An optical image showing the strip device to power an LED bulb by harvesting body heat with the assistance of a voltage amplifier

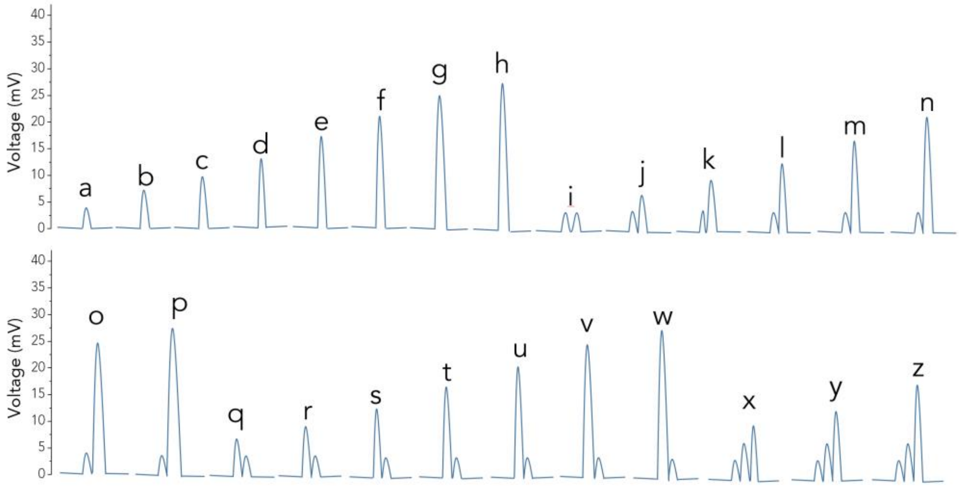

Fig. S14 Depiction of the distinct voltage signals for the 26 letters

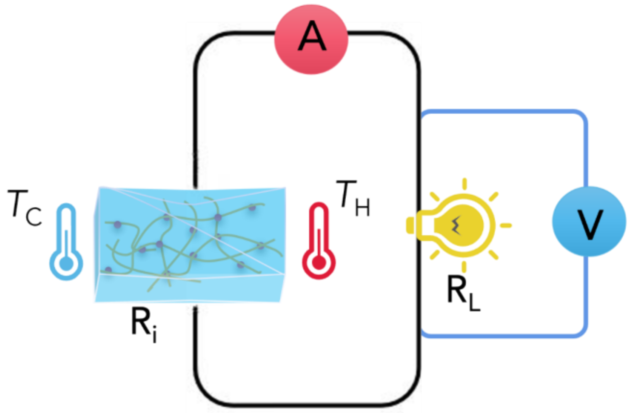

Fig. S15 Illustration of the connecting circuit of the self-powered TEC motion sensor

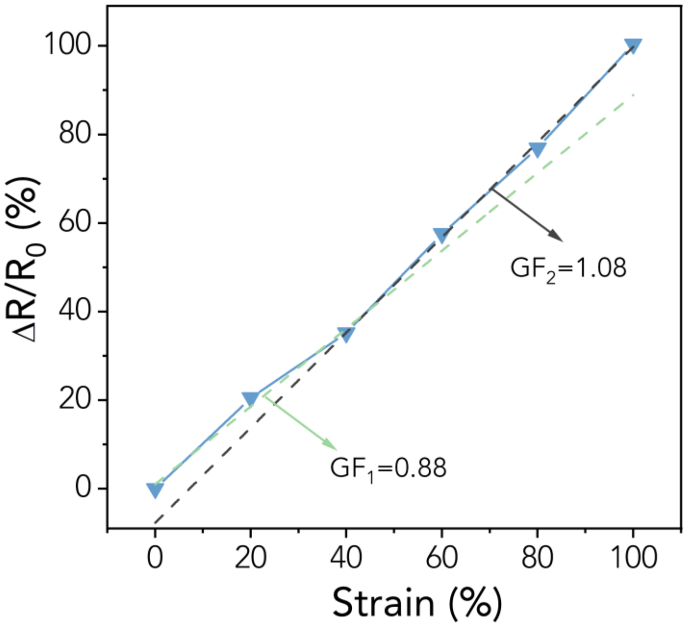

Fig. S16 The GF value for the TEC sensor in the strain range of up to 100%

The variation in the *GF* value within the two linear regions can potentially be attributed to the tunneling effect and the contact-resistance effect. Under conditions of small strain, the MXene nanosheets manage to sustain a relatively stable conductive network, despite experiencing an increase in both tunnel and contact resistance. However, as the strain intensifies, the nanosheets tend to become more disarrayed, or in some instances, completely disconnected. This significant disruption to the nanosheets' organization substantially impedes the conductive pathways, thereby instigating a pronounced escalation in electrical resistance.

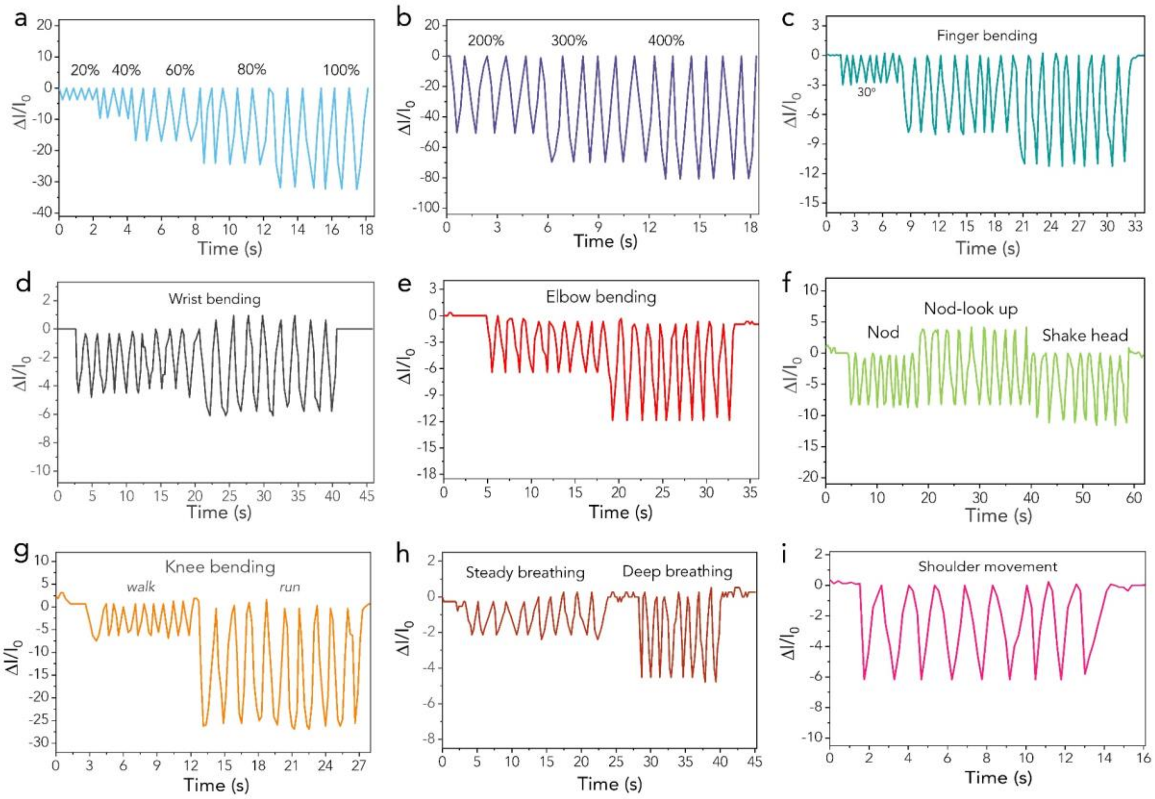

Fig. S17 The variation in current ($\Delta I/I_0$) with strain under various body motions

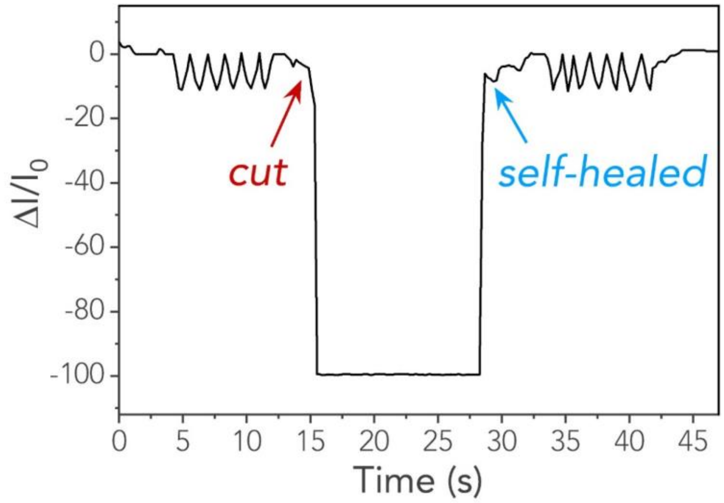

Fig. S18 The $\Delta I/I_0$ signal before and after a cut-healing process
